# Effects of crowding and sex on fecal cortisol levels of captive forest musk deer

**DOI:** 10.1186/0717-6287-47-48

**Published:** 2014-09-29

**Authors:** Lan He, Wen-Xia Wang, Lin-Hai Li, Bao-Qing Liu, Gang Liu, Shu-Qiang Liu, Lei Qi, De-Fu Hu

**Affiliations:** Laboratory of Non-invasive Research Technology for Endangered Species, College of Nature Conservation, Beijing Forestry University, No. 35 Tsinghua East Road, Haidian District, Beijing, 100083 China; Key Laboratory of Species Diversity Application and Control in Xinjiang, Urumqi, Xinjiang Normal Univesity, Xinjiang, 830054 China; Breeding Centre of Forest Musk Deer in Pientzehuang, Baoji, 721000 China; Xinjiang Normal University, NO.102, the Xinyi Road, Urumqi, Xinjiang, 830054 China; Beijing Natural History Museum, No.126, the South Street, Dongcheng District, Beinjing, 100050 China

**Keywords:** Captivity, *M. berezovskii*, Fecal cortisol, Chronic stress, Crowding

## Abstract

**Background:**

Restricted space and close contact with conspecifics in captivity may be stressful for musk deer, as they are highly territorial and solitary in the wild. So we tested the effects of crowding on stress of forest musk deer (*Moschus berezovskii*) in heterosexual groups, using fecal cortisol analysis as a non-invasive method. 32 healthy adults during non-breeding seasons were chose as our experimental objects. Group 1 was defined as higher crowding condition, with 10-15 m^2^/deer (6 enclosures, 10♀ and 6♂); group 2 was defined as lower crowding condition, with 23-33 m^2^/deer (6 enclosures, 10♀ and 6♂). Every enclosure contained 1 male and 3 female. These patterns had been existed for years.

**Results:**

The results showed that females in lower crowding condition (217.1 ± 9.5 ug/g) had significantly higher fecal cortisol levels than those in higher crowding condition (177.2 ± 12.1 ug/g). Interestingly, crowding seemed have no effect on male fecal cortisol levels (148.1 ± 9.1 ug/g and 140.5 ± 13.3 ug/g, respectively). At both groups, cortisol was significantly lower in males than in females.

**Conclusions:**

These results showed that chronic crowding may affect stress status of captive forest musk deer. The captive environment should consider the space need for musk deer.

## Background

Captive breeding is an important tool for conservation of endangered wild animals. However, potential stressors in the captive environment are numerous, and their effects are often species-specific [1, 2]. Captive animals are likely suffering chronic stress [3], resulting from lack of space, abnormal social groups and/or other restrictions to the animals’ natural behavioral needs [4]. Chronic stress may cause physiological and psychiatric disorders [5, 6], and decreased immunity [7, 8]. So it’s very important to assess and identify the main stressors for effective population management and the ultimate success of captive breeding programs.

Musk deer (*Moschus* spp.) are small solitary ungulates distributed throughout forested and mountainous parts of Asia. They are world-famous because male musk deer secrete musk from the musk gland, located between their naval and genitals [9–11]. Musk has been used in Chinese traditional medicine for over 2000 years, and use in expensive perfume material in the European countries due to its permanent and special scent. Currently, captivity has become the main methods to conserve musk deer, since their wild population is almost exhausted. However, their captive populations grow slowly. Although it’s generally considered that musk deer is difficult to be captive because of their timid and alert characteristics, these characteristics easily cause stress responses. But there are yet no studies about relationships between captive environment and stress status of musk deer. Since 2003, China drawn up “*the Conservation Plan of Musk deer*”, in which, establishing large scale of captive population is one of the main objects. Undoubtedly, lack of this information will affect the establishing of breeding population and formulating reasonable measures of the management.

Crowding is a common stressor in captive environment [4]. Crowding (means restricted space and close social contact) may be much stressful for musk deer, since they are solitary and territory animals in the wild; however no relative experiments have been reported.

Here, we used a non-invasive method to test the relationship between captive environment and stress status of forest musk deer (*M. berezovskii*), one of the main captive species of musk deer in China, in order to help understanding the reason of difficult population development in musk deer, and improving welfare and conservation of these animals.

The objectives of this study were to (1) test whether different crowding condition (which means restricted space and close contact with conspecifics) affect fecal cortisol concentration (FCC) of musk deer; (2) test whether female and male musk deer response differently to crowding conditions, when they are reared in heterosexual groups.

## Results

Only 4 individuals had extreme FCC that was 2SD above baseline, two were the female in group 1, one was the female in group 2, and one was the male in group 2 (Tables 1 and 2), these values were excluded and baselines were recalculated. The FCC fluctuated, but most values were within 95% confident interval. The ranges of FCC changed from 23.9 to 101.3 ug/g, 25.8 to 114.9 ug/g for females reared in higher and lower crowding conditions, respectively (Table 1). And the FCC ranges changed from 34.0 to 70.8 ug /g, 65.9 to 96.4 ug/g for males reared in higher and lower crowding conditions, respectively (Table 2).

**Figure 1:**
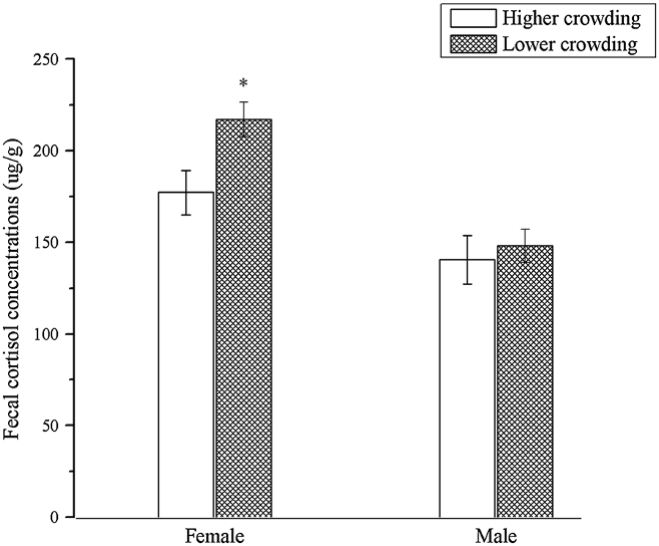
**Fecal cortisol levels, standard errors of female (n = 20) and male (n = 12) musk deer in different crowding conditions.** Females had significantly higher cortisol levels in lower crowding condition than in higher crowding condition (P < 0.05), but males had similar cortisol levels (P > 0.05). Cortisol concentrations were significantly lower in males than in females at both crowding conditions (P < 0.05).

For the mean baseline values, FCC of females reared in group 1 was 177.2 ug/g, which was significantly lower than that of the group 2, 217. 1 ug/g (Figure 1) (t = -2.599, P = 0.018). But for males, FCC were 148.1 ug/g and 140.5 ug/g in group 1 and group 2 respectively, without significant differences (Figure 1) (t = 0.468, P = 0.650).

There was significantly differences when take sex into account. FCC of females was 19.9% and 54.5% higher than that of the male musk deer reared in higher and lower crowding conditions, respectively (t = 2.719, P = 0.017; t = 4.794, P = 0.000) (Figure 1).

**Table 1:** **The highest and lowest value, variation range, and standard errors of female FCC (n = 20)**

	Musk deer	Highest FCC (ug/g)	Lowest FCC (ug/g)	Variation range of FCC (ug/g)	Standard error
Higher crowding	1	145.6	121.7	23.9	3.0
	2	179.1	128.9	50.2	6.3
	3	273.7*	177.6	96.1	12.2
	4	268.4*	190.2	78.2	23.3
	5	186.3	133.8	52.5	6.1
	6	236.8	135.5	101.3	12.0
	7	193.8	153.9	39.9	5.6
	8	232.4	167.2	65.2	9.1
	9	226.2	169.6	56.6	8.2
	10	211.7	170.8	40.9	6.3
Lower crowding	1	239.4	165.6	73.8	9.5
	2	319.9*	208.4	111.5	14.0
	3	296.5	181.6	114.9	14.2
	4	206.8	181.0	25.8	4.0
	5	249.7	185.4	64.3	8.3
	6	252.4	175.6	76.8	10.6
	7	265.9	169.9	96.0	12.9
	8	285.5	194.4	91.1	12.0
	9	241.8	195.8	46.0	6.5
	10	230.8	175.7	55.1	7.3

Note: *represent values that 2SD above the baseline.

**Table 2:** **The highest and lowest value, variation range, and standard errors of male FCC (n = 12)**

	Musk deer	Highest FCC (ug/g )	Lowest FCC (ug/g)	Variation range of FCC (ug/g)	Standard error
Higher crowding	1	157.1	123.1	34.0	8.5
	2	180.0	116.0	64.0	7.9
	3	166.5	122.2	44.3	6.8
	4	184.8	134.4	50.4	6.6
	5	179.6	108.8	70.8	9.6
	6	187.7	125.4	62.3	8.9
Lower crowding	1	181.5	109.0	72.5	11.9
	2	180.7	114.8	65.9	7.8
	3	194.3	110.9	83.4	10.2
	4	192.8	114.3	78.5	12.6
	5	170.9	94.3	76.6	9.5
	6	212.3*	115.9	96.4	12.3

Note: *represent values that are 2SD above the baseline.

## Discussions

### Effect of chronic crowding stress

Captive environment is much different than wild environment, thus it would potentially stressed captive animals. There are many stressors in captive environment [4], and stress effect may be existing even when animals are reared in captivity for many generations [12]. Crowding is one of the most important stressors in captive environment, which means less space and close contact with conspecifics. Most studies reported higher glucocorticoid levels according to crowding. For example, plasma cortisol levels of non-human primate in crowding groups were significantly higher than paired individuals [13–15]. Li et al. [16] found that changes from large enclosures to small pens resulted in higher level of cortisol secretion in Pe’re David’s deer (*Elaphurus davidianus*).

However, in this study, compared with less crowding condition (217.1 ug/g), female musk deer housed in higher crowding condition (177.2 ug/g) had significantly lower FCC (Figure 1). These inconsistent glucocorticoid responses to crowding may due to different stress time, and different personality of species—different species may responses to the same stressors differently [1, 2]. Forest musk deer are territory, solitary, shy, and timid mammals in the wild [11, 17]. However, in captivity, they are compelled to be housed with each other in an enclosure that is 40 to 132m^2^. No doubt that the increased social contact and decreased space were stressful for such solitary and shy animals. What’s more, forest musk deer like jumping and running, that’s their survival strategies evolved in the wild. However, the captive space is too small to exhibit this behavior, and they developed stereotyped behavior (such as repeated walk back and forth, constant jump up and down). Our investigation in other breeding centers show that forest musk deer in semi-free captive environment (more natural environment, larger space that is about 660 m^2^ and about 200-300 m^2^ per deer) have significantly lower stereotyped behavior than those in captive environment (certainly, the stereotyped behaviors did not disappeared). And we found that, adults in semi-free captive environment have less disease, such as diarrhea, dyspepsia, and abscess disease (unpublished data). Liu et al. [18] also reported that diseases of forest musk deer decreased when they were reared in natural enclosures (about 400 m^2^). In this study, the rearing pattern is one male with three female musk deer, the decreased cortisol levels of musk deer might be the response to close contact with conspecifics and lack of home range, which might become chronic stress as time passed.

Miller et al. [19] reviewed that chronic stress will cause both increase and decrease of glucocorticoid levels. Shortly after the stress has begun, the hypothalamic-pituitary-adrenal axis may become activated, resulting in elevated corticoid output. However, with the passage of time, the body could mount a counter-regulatory response such that corticoid output rebounds below normal [19]. Recently, many researchers found the decreased glucocorticoid levels in captive or wild animals. For example, in free-living and wild-caught European starlings (*Sturnus vulgaris*) exposed to an experimental chronic stress consisting of unpredictable, and different rotating stressors, both baseline and stress-induced corticosterone are suppressed [20, 21], and sensitivity of the pituitary and adrenal gland are altered [19]. Linklater et al. [22] also suggested that captivity results decline of fecal corticoid levels in rhinoceros after translocation. In our study, the decreased cortisol levels of the captive forest musk deer may reflect the end stage of stress—because all of the experimental musk deer have been captive reared since they were born, such long time may bring them into the end status of the chronic stress.

An interesting but inexplicable phenomenon is that crowding seems don’t affect stress of male forest musk deer, because males in both groups had similar FCC. One possibility is that male musk deer are highly stressed under captive environment, which might cover the crowding effect. Much more has been discussed below.

### Sex difference of cortisol levels in heterosexual groups

In our study, crowding environment means less space and close social contact with conspecifics. Animals cope with social contact differently according to group type, group size, and gender. Our study show an apparently different cortisol levels in different sexes, males had significantly lower FCC than females (19.9% and 54.5% higher in female than in male). Sex differences in adrenocortical activity are not uncommon in other mammals. For example, female North American clouded leopards (*Neofelis nebulosa*) and female mouse lemur (*Microcebus murinus*) had significantly higher corticoid levels than males [15, 23]. Other studies about glucocorticoid levels in rodents and humans according to gender are inconsistent. For example, adrenal weight is greater in the male hamster than in the female [24]. However, other studies about rats showed that corticosterone concentration were higher in female than male rats [25, 26]. These sex differences may due to different gonadal hormone effect [27–30], and glucocorticoid receptor and binding protein levels [30, 31]. The sex differences may reflect underlying differences in steroid metabolism, excretion routes, and pituitary responsiveness [32].

However, in this study, the sex differences about cortisol levels in forest musk deer might reflect different stress status. Many studies about rodent and humans show that the female can influence male’s behavior and physiology [33, 34]. Naturally, musk deer are solitary, even the female and male musk deer encounter and stay with each other briefly only during breeding seasons, and they separate and return to their own territories as soon as mating succeeded/ended [11]. In captivity, several male forest musk deer are reared together with several females in an enclosure (in our study, the rearing pattern is one male with three female), all the year round. Such unnatural environment may cause chronic stress; female musk deer may enhance the stress of male individuals. Our investigation show that, when the male and the female forest musk deer are reared separately during the non-breeding season—several males live together in an enclosure without see each other (everyone has an individual house), and are allowed by turns to play and move in the outside yard. Their fecal cortisol levels are similar with females at the non-breeding seasons. So it could be speculated that, the long-term exist of female musk deer might enhance the stress of males, leads to lower cortisol levels in the males. It seems to explain, at least part of, the reasons of higher mortality in the male than in the female forest musk deer ([35], and our unpublished data). And during the mating seasons, many centers lack the seed breeding male forest musk deer (our surveys), which might be the results of chronic captive stress. Fortunately, in recent years, some centers began semi-free rearing practices, which may improve welfare of musk deer.

However, other ungulates, such as red deer (*Cervus elaphus*), did not found significant differences between sexes [36]; but they performed the study on an undisturbed red deer herd, kept in a 45-ha enclosure. While in our study, the forest musk deer are captive in an environment with restricted space (only less than 132m^2^). What’s more, red deer is social species, while forest musk deer is solitary animals. In the captive environment, however, they are compelled to live with conspecifics. Such unnatural environments may induce highly stress.

It’s a pity that we did not collect wild feces of forest musk deer, and we do not know the actual stress status of forest musk deer in the wild. So the relationship of stress status of forest musk deer between the captive and wild conditions should be further studied. Furthermore, although female musk deer lived in lower crowding condition had higher cortisol levels, but we could not determine that these individuals are comfortable. It’ should be pointed out that in this study, we defined the crowding degree as relative higher and lower, because we yet do not know the threshold of un-crowding conditions.

## Conclusions

In sum, we suggest that crowding condition may affect stress of forest musk deer. Though we yet do not know which the best environment for captive musk deer is, but it’s certain that too small captive environment is not suitable for the welfare of musk deer. The current enclosure designs may be too small to satisfy their natural need. And we also suggest that, the rearing patterns of heterosexual group might not be appropriate, long-term of stimulation by the females might enhance stress of males. So the future management should consider the space need and the group component.

## Methods

### Study site and animals

The study was conducted at Breeding Center of Forest Musk Deer, located in Fengxian, Shanxi Province, a region of Qing Ling Mountain (33°-34°N, 106°-107°E). The region is in a warm temperate zone, with an annual average temperature of 11.4°C and annual average rainfall of 613.2-897.1 mm.

From April to September is the non-breeding season [11, 37]. All animals were fed twice per day, at dawn and dusk, with fresh leaves (in summer and autumn) or dried leaves (in winter and spring), which were collected from the natural habitat of wild musk deer. The plants include nutgall (*Anacardiaceae rhus*), Chinaberry seed (*Simaroubaceae picrasma*), elm (*Ulmus pumila*), etc. Supplementary artificial food mainly consisted of flour, wheat bran and some seasonal vegetables. Water was provided *ad libitum*.

One male with three female musk deer (*Moschus berezovskii*) were kept in an enclosure which consisted of an outdoor yard, and lined several brick houses, the total area of each enclosure is about 40-132m^2^. All the male and females in an enclosure can move and play in the yard during the day; they were kept individually in each individual house during the night. These group patterns have been exist for years in this breeding center.

We define the average space of 10-15m^2^ per individual as group1 (higher crowding condition), and the average space of 23-33m^2^ per individual as group 2 (lower crowding condition). The two groups contains 6 enclosures respectively, each group contain 6 males and 10 females. All the animals were healthy adults aged 3-7 years, and both were captive, without wild-caught animals.

### Sample collection

The samplings were conducted during August to September in 2011. This period is non-breeding season, although the farmers reared the female and male forest musk deer together as in breeding season. This period also is the period of delactation (offspring are separated from mothers). We chose this period to avoid other stressors, such as mating, rearing and protecting offspring.

Feces were collected every other day for each subject for two weeks. Every afternoon at 19:00-20:00 P.M, the raiser cleaned feces out of the individual house, so we can collect fresh feces (defecate during the night) for each individual at the next morning. The feces were collected at 6:00 to 8:00 A.M. and stored frozen at -20°C immediately after collection. The Sampling collection was carried out by the raiser instead of the researchers in order to decrease stress response.

The fecal sampling (non-invasive method) was carried out under the authority of a scientific permit issued by Shanxi Forestry Bureau, Shanxi, China. Our sampling method is non-invasive, that we only collected animal's feces.

### Fecal cortisol extraction and determination

Fecal cortisol was extracted as previously described with little changes [37]. Briefly, frozen samples were thawed at the room temperature, and the feces were homogenized in a grinder. Then, 0.5 g of feces was mixed with 5 ml of 90% methanol in a 15-ml glass tube and extracted using a “water bath” at 60°C for 20 min. All tubes were then centrifuged at 2500 rpm for 20 min and the supernatant was recovered. An additional 5 ml of 90% methanol was added to the fecal pellet, which was then vortexed for 1 min. and centrifuged at 2500 rpm for 15 min. The supernatants were recovered. The combined supernatants were dried, re-dissolved in 1 ml methanol and the solution was kept at −20°C until assayed.

Fecal cortisol quantitative diagnostic kits were obtained from Shanghai Yueyan Biological Technology Co., Ltd., Shanghai, China. The assays were performed according to the manufacturer’s directions. Sensitivity parameters are as followers, sensitivity is ≤ 5.0 ug/ml, intra-assay CV is <7% and the inter-assay CV is <8.5%, no cross reaction.

For each feces, water content was calculated (dry in the oven at 65°C for 8 h) and was used to adjust the final fecal cortisol concentrations (FCC), so the unit of the FCC was transformed from ug/ml to ug/g.

### Data analysis

FCC is reported as the baseline mean ± standard error of the mean. Individual baseline means were calculated for each animal using an iteration process where all peak values two standard deviations above the mean were excluded and means were recalculated until extreme values were excluded [38].

Assumptions of normality were checked by examining normal probability plots and calculating a Kolmogorov-Smirnov statistic. An independent-samples t-test was used to analyze sex and crowding effects. For all analyses, significance was set at the 0.05 level. Statistical analyses were conducted using SPSS Version 18.0 for Windows. Figure was drawn using Origin 8.0.
